# From Trauma to Tumour: Incidental Giant Left Adrenal Myelolipoma in Congenital Adrenal Hyperplasia

**DOI:** 10.7759/cureus.117583

**Published:** 2026-10-07

**Authors:** Emily M Mayar, Anna Crown, Sing Y Sim

**Affiliations:** 1 Endocrinology, University Hospitals Sussex NHS Foundation Trust, Brighton, GBR

**Keywords:** 21-hydroxylase deficiency, acth hypersecretion, adrenalectomy, adrenal incidentaloma, adrenal myelolipoma, congenital adrenal hyperplasia

## Abstract

Adrenal myelolipomas are rare, benign tumours composed of mature adipose and haematopoietic tissue. Patients with congenital adrenal hyperplasia (CAH) are predisposed to their development, with chronic adrenocorticotropic hormone (ACTH) hypersecretion secondary to impaired cortisol synthesis thought to be the principal driver of myelolipoma formation in this population. A 49-year-old female with known CAH presented to the emergency department following a stab wound to the abdomen. Computed tomography (CT) incidentally identified a 12 cm encapsulated left adrenal lesion consistent with a giant myelolipoma. Despite reported adherence to hydrocortisone and fludrocortisone replacement therapy, persistently elevated adrenal steroid precursors and plasma renin levels suggested suboptimal glucocorticoid control. Following multidisciplinary team (MDT) review, the patient was offered a left adrenalectomy given the lesion size (>10 cm) and concerns regarding the risk of spontaneous haemorrhage. This case underscores the importance of systematic imaging review beyond the primary indication for scanning, and the value of optimising biochemical monitoring and considering adrenal imaging in selected high-risk CAH patients.

## Introduction

Adrenal myelolipomas are rare, benign tumours composed of mature adipose tissue and haematopoietic elements, with an estimated prevalence of 0.08-0.25%, accounting for approximately 3-5% of all primary adrenal tumours [1]. Most present as incidentalomas, with increasing detection attributed to the widespread use of cross-sectional imaging [2]. The majority are asymptomatic and managed conservatively; however, giant myelolipomas exceeding 10 cm carry a clinically significant risk of spontaneous haemorrhage [2].

Patients with CAH have a myelolipoma prevalence of 7.4%, with the vast majority of those affected (93.5%) having undiagnosed or inadequately controlled disease [3]. Impaired cortisol synthesis drives chronic ACTH hypersecretion, stimulating adrenocortical hyperplasia which may undergo metaplastic transformation into haematopoietic tissue [4], and this chronic ACTH-driven mechanism is considered central to the disproportionate burden of myelolipoma observed in patients with inadequately controlled CAH [3,4]. 

Nevertheless, adrenal myelolipomas remain uncommon even among patients with CAH, and current Endocrine Society guidelines do not recommend routine imaging surveillance in asymptomatic CAH patients [4].

We report a giant incidental adrenal myelolipoma in an adult with classical CAH and prolonged biochemical evidence of inadequate disease control, highlighting the interplay between chronic hormonal stimulation and our surgical decision-making.

This case was previously presented as a poster at the European Congress of Endocrinology in the Czech Republic on 9-12 May 2026.

## Case presentation

A 49-year-old female with a confirmed diagnosis of classical CAH due to 21-hydroxylase deficiency (21-OHD) presented to the emergency department following a stab wound to the abdomen. She had been diagnosed at birth following the identification of ambiguous genitalia. Her brother carries the same diagnosis and has developed bilateral adrenal myelolipomas. Her background medical history was otherwise unremarkable.

On presentation, she was haemodynamically stable. Emergency CT of the abdomen and pelvis confirmed a superficial abdominal wall injury with no evidence of intra-abdominal penetrating injury. Incidentally, imaging demonstrated a 12 cm encapsulated left adrenal lesion containing fat, soft tissue, and calcification, with appearances consistent with a giant adrenal myelolipoma (Figure 1). An additional 10 mm right adrenal nodule was also noted incidentally, measuring 17 HU on unenhanced CT.

**Figure 1:**
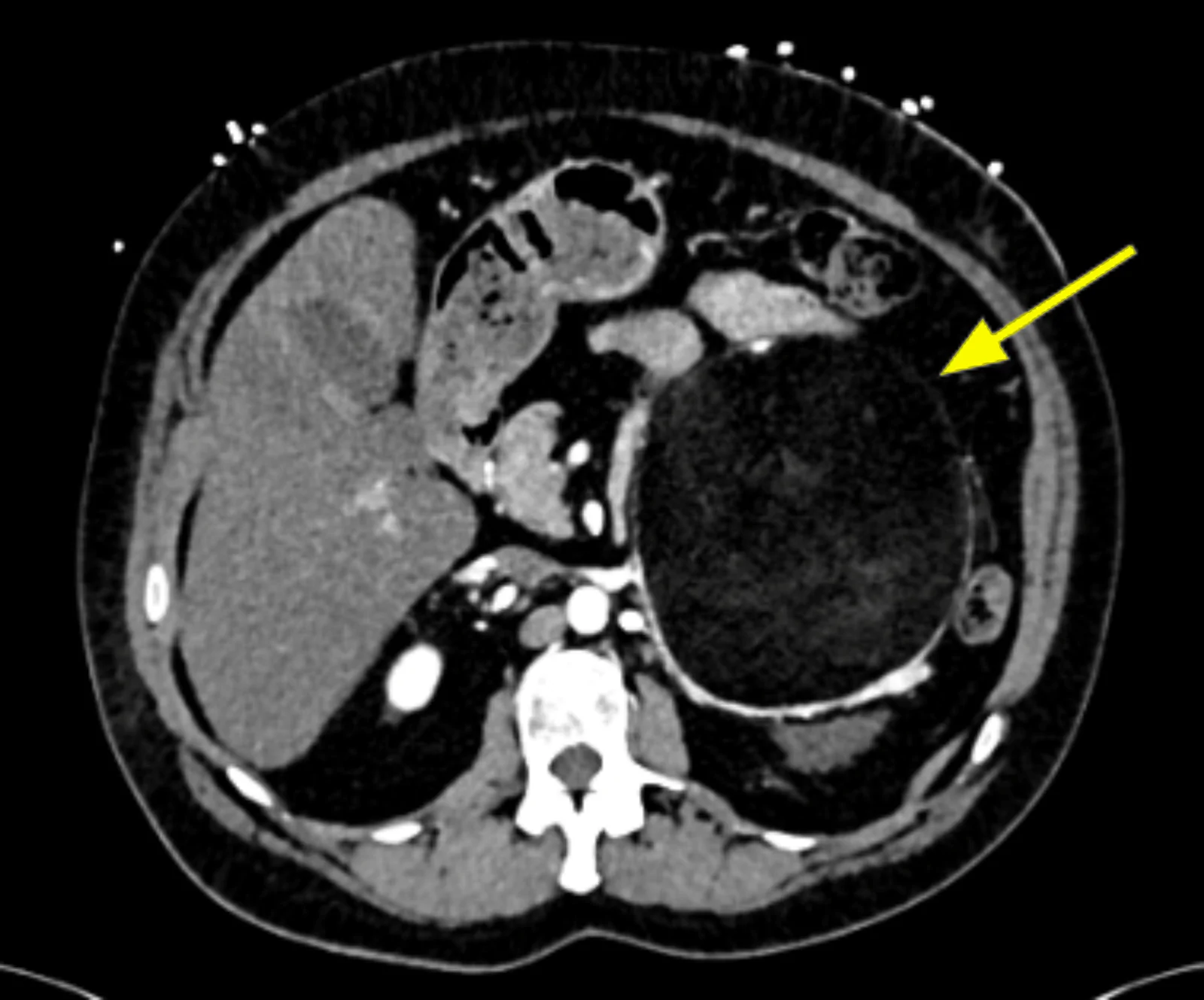
Transverse CT views of the abdomen demonstrating the incidental 12 cm encapsulated left adrenal lesion containing fat, soft tissue, and calcification, consistent with a giant adrenal myelolipoma

The patient was reviewed in the endocrine outpatient clinic and reported adherence to her prescribed replacement regimen of hydrocortisone 10 mg twice daily and fludrocortisone 200 micrograms daily. However, markedly elevated androgen and mineralocorticoid markers indicated likely suboptimal glucocorticoid and mineralocorticoid replacement, whether due to non-adherence, inadequate dosing, or both (Table 1).

**Table 1:** Biochemistry at presentation, demonstrating markedly elevated androgen and mineralocorticoid markers consistent with suboptimal glucocorticoid and mineralocorticoid replacement eGFR: estimated glomerular filtration rate

Parameter	Result	Reference Range
17-Hydroxyprogesterone	117.5	1.0–8.7 nmol/L
Androstenedione	18.8	2.0–5.4 nmol/L
Renin	191.0	5.4–60.0 mU/L
Testosterone	4.3	0.29–1.67 nmol/L
Sodium	135	136–145 mmol/L
Potassium	4.9	3.5–5.1 mmol/L
eGFR	>60	60–90 mL/min/1.73m²

A longitudinal trend in 17-hydroxyprogesterone (17-OHP) was available from previous clinic attendances and is presented in Table 2. Persistently elevated values over more than a decade indicate that disease control has been chronically suboptimal.

**Table 2:** Longitudinal 17-hydroxyprogesterone trend, demonstrating persistently elevated values over more than a decade, consistent with chronically suboptimal glucocorticoid replacement.

Date	17-OHP (nmol/L)	Reference Range (nmol/L)
December 2013	107.0	0–10
January 2014	44.0	0–10
August 2016	131.0	0–10
July 2025	117.5	0–10

Long-term management of classical CAH requires balancing androgen suppression against the risks of iatrogenic glucocorticoid excess. Recognising that the patient was comfortable with her phenotype provides context for both her biochemical data and her lapse in clinic attendance. In this patient, maintaining an androgen exposure profile aligned with a gender phenotype she was content with, combined with a complete absence of adrenal crisis, shaped her engagement with therapy over time.

Following adrenal MDT review, a left adrenalectomy was recommended given the lesion size (12 cm) and concerns regarding the risk of spontaneous haemorrhage [2,5]. At the time of writing, the patient is awaiting surgical intervention. Postoperative follow-up will include biochemical reassessment of androgen and mineralocorticoid markers and ongoing optimisation of replacement therapy. Imaging follow-up of the right adrenal nodule is also recommended given its indeterminate attenuation of 17 HU on unenhanced CT, to monitor for growth or malignant features in keeping with current ESE guideline recommendations [6].

## Discussion

This case illustrates several clinically important themes relevant to the long-term management of CAH. Firstly, the incidental discovery of a giant adrenal myelolipoma during a trauma CT presentation underscores the value of systematic review of all imaging findings, irrespective of the primary presenting complaint.

In CAH, chronic ACTH hypersecretion, driven by insufficient glucocorticoid-mediated negative feedback, stimulates adrenocortical hyperplasia, which may undergo metaplastic transformation into haematopoietic tissue, culminating in myelolipoma formation [4]. Patients with CAH have a myelolipoma prevalence of 7.4%, with the vast majority of those affected (93.5%) having undiagnosed or inadequately controlled disease [3]. 21-OHD is inherited in an autosomal recessive pattern, with greater ACTH-driven adrenal pathology observed in those with severe salt-wasting or simple virilising forms of the condition [4,7]. While evidence-based clinical guidelines recommend against routine adrenal imaging screening in CAH [4], in clinical practice, targeted imaging may be considered on an individualised basis in selected cases such as those with an established lesion. When monitoring is considered, the optimal modality, frequency and cost-effectiveness remain undefined. CT introduces cumulative ionising radiation risks; ultrasound eliminates radiation exposure but is operator-dependent and would not detect smaller adrenal lesions; unenhanced MRI is an alternative though resource availability varies. 

The persistently elevated 17-OHP, androstenedione, testosterone, and renin levels spanning more than a decade despite reported adherence to replacement therapy suggest that glucocorticoid and mineralocorticoid control has been chronically suboptimal. Whether this reflects true non-adherence, inadequate dosing, pharmacokinetic variability, or a combination of factors warrants careful clinical assessment [4]. Persistently elevated androgen and mineralocorticoid markers should prompt assessment of both medication adherence and adequacy of glucocorticoid and mineralocorticoid replacement dosing. Importantly, ongoing ACTH hypersecretion may have perpetuated progressive tumour growth, and optimisation of replacement therapy may represent a modifiable factor in reducing the risk of further adrenal pathology. However, prospective evidence demonstrating that optimising biochemical control halts growth or induces regression of established lesions remains lacking.

Giant myelolipomas (>10 cm) are frequently managed surgically due to concerns regarding spontaneous rupture. Retroperitoneal haemorrhage, should it occur, may result in haemodynamic instability necessitating emergency intervention [1,2]. However, the exact annual incidence of haemorrhage across different size thresholds remains unknown, as data are limited primarily to retrospective case series. Surgical decision-making therefore relies on expert consensus rather than precise risk quantification [2,5]. This case reinforces that myelolipoma size and biochemical disease control should both be integrated when stratifying surgical risk and planning intervention. Multidisciplinary management is essential for operative planning, perioperative steroid cover, and monitoring of the contralateral adrenal gland [4].

## Conclusions

This case illustrates the importance of opportunistic surveillance for adrenal pathology in all CAH patients, regardless of the presenting complaint. Biochemical monitoring should be maintained in long-term CAH follow-up; whether routine adrenal imaging improves outcomes in this population remains to be established. Future research should determine whether targeted adrenal imaging in high-risk CAH patients facilitates earlier detection of clinically significant adrenal myelolipomas and, if so, establish the most appropriate imaging modality, surveillance interval and cost-effective strategy. Optimising glucocorticoid and mineralocorticoid treatment is critical for hormone replacement, control of adrenal androgen production and reduction of ongoing ACTH-driven adrenal growth. This case serves as a reminder that incidental adrenal pathology must never be overlooked, even in the context of an acute trauma presentation.
